# Symphony of the gut microbiota and endocannabinoidome: a molecular and functional perspective

**DOI:** 10.3389/fcimb.2025.1566290

**Published:** 2025-03-26

**Authors:** Yang Wang, Jing Guo, Zhiqin Mao, Ying Chen

**Affiliations:** Department of Pediatric Gastroenterology, Shengjing Hospital of China Medical University, Shenyang, Liaoning, China

**Keywords:** endocannabinoid system (ECS), endocannabinoidome (eCBome), gut microbiota, symphony, interactions

## Abstract

This review examines the impact of interactions between the gut microbiota and the endocannabinoidome (eCBome) on health and disease, highlighting their significance for physiological and pathological processes. We identify key research gaps and challenges to advance the field. The review discusses the role of dietary patterns and physical activity in regulating these interactions. It also explores the complex nature of these interactions in conditions such as inflammatory bowel disease (IBD), depression, anxiety, Alzheimer’s disease (AD), and metabolic disorders. This analysis evaluates their contributions to disease onset and progression, and examines the molecular mechanisms and signaling pathways involved. From this, we provide forward-looking perspectives on future research directions, advocating for a more nuanced understanding of the gut microbiota–eCBome axis. We anticipate that future research will integrate gut microbiota–endocannabinoidome interactions into therapeutic strategies for a broad range of diseases.

## Introduction

The gut microbiota, consisting of approximately 100 trillion microorganisms, colonizes the intestines at birth and dynamically adapts to environmental factors. The microbiota includes not only bacteria but also viruses, fungi, archaea, and eukaryotes, together forming our “second genome” (Bäckhed et al., 2005). Seven major bacterial phyla play crucial roles in the gut, with Firmicutes and Bacteroidetes being the most dominant. Firmicutes, which are primarily Gram-positive bacteria, and Bacteroidetes, composed mainly of Gram-negative anaerobes, are key players in maintaining gut health. A balance between these phyla is essential for maintaining gut health (Islam et al., 2022).

The gut is divided into four sections: the duodenum, jejunum, ileum, and colon. Each section has distinct microbial densities and compositions. The duodenum has the lowest bacterial density, predominantly consisting of Firmicutes and Actinobacteria. In contrast, the colon, with the highest bacterial density, is dominated by Firmicutes and Bacteroidetes. Key colon genera include Bacteroides, Bifidobacterium, and Lactobacillus (Hollister et al., 2014). The gut microbiota influences host health on multiple levels, such as metabolism, immune protection, structural integrity, and neural regulation (Adak and Khan, 2019). Through host interactions, these microbes regulate physiological processes and are indispensable for maintaining overall health.

The endocannabinoid system (ECS) is a complex network composed primarily of the endocannabinoid molecules anandamide (AEA) and 2-arachidonoylglycerol (2-AG), along with their corresponding cannabinoid receptors 1 and 2 (CB1 and CB2). The system also includes key enzymes like N-acyl-phosphatidylethanolamine-specific phospholipase D-like and diacylglycerol lipases α and β, as well as degradative enzymes like fatty acid amide hydrolase (FAAH) and monoacylglycerol lipase. AEA and 2-AG belong to the N-acylethanolamine (NAE) and monoacylglycerol (MAG) families, respectively. The ECS plays critical roles in regulating processes such as pain perception, appetite, and mood regulation (TantiMonaco et al., 2014; Lowe et al., 2021).

**Figure 1:**
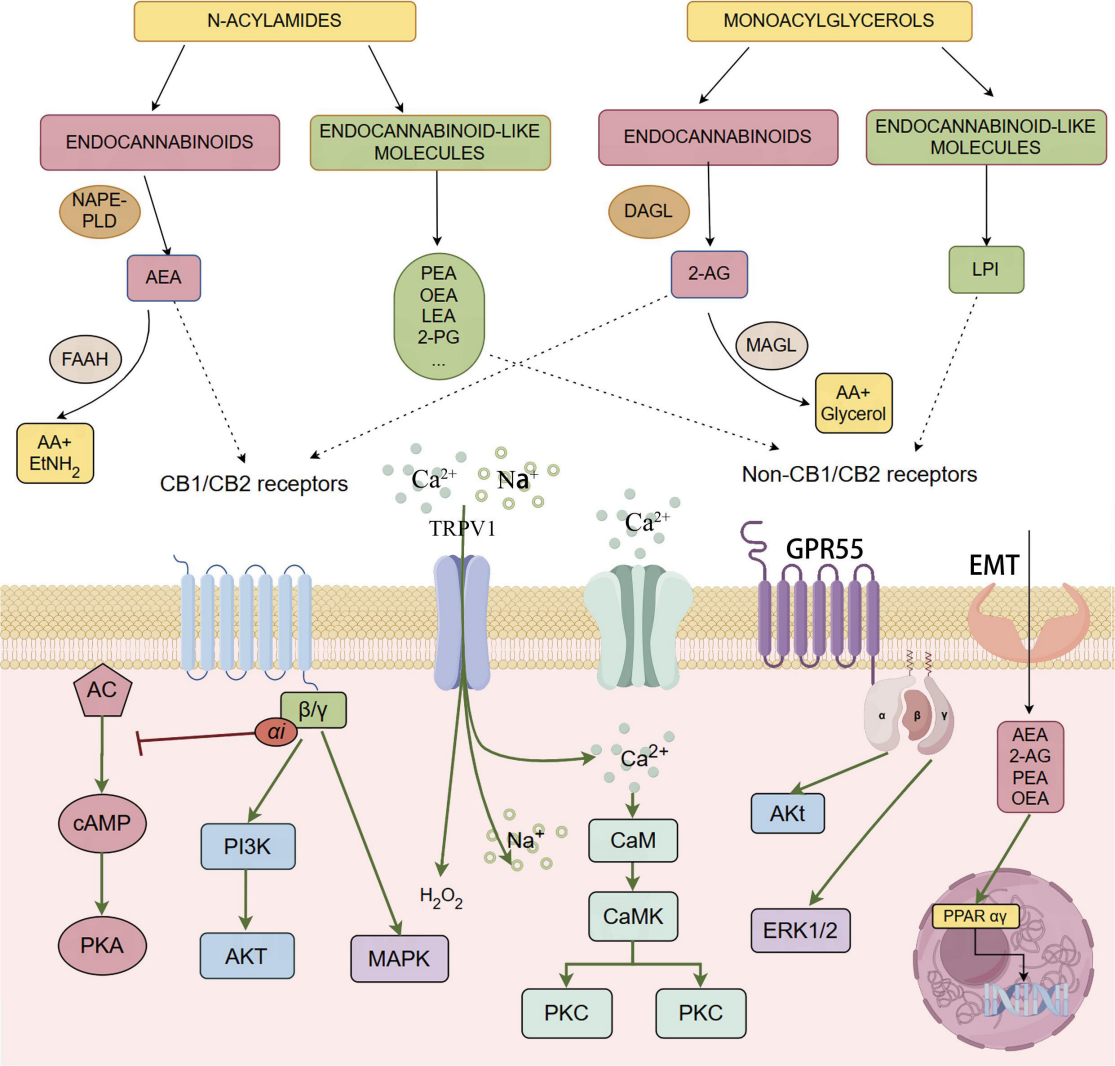
The figure provides a comprehensive overview of the endocannabinoidome, including both the classical endocannabinoid system and its extended network of molecules and receptors, such as CB1, CB2, GPR55, and PPARs. Endocannabinoids like AEA and 2-AG bind to these receptors, regulating neural and immune signaling pathways through the activation of G-proteincoupled receptors and the modulation of second messengers such as cAMP and Ca2+. Additionally, the figure illustrates the synthesis and degradation mechanisms of endocannabinoids and related lipid molecules, highlighting their broad regulatory roles in various physiological processes.

Ongoing research has expanded the ECS into a broader system, the endocannabinoidome (eCBome), which includes additional lipid mediators and receptors. These include molecules such as palmitoylethanolamide (PEA), oleoylethanolamide (OEA), stearoylethanolamide, linoleoylethanolamide (LEA), N-docosahexaenoylethanolamine, 2-oleoylglycerol (2-OG), 2-palmitoyl-glycerol, 2-linoleoyl-glycerol, and others. These molecules share biosynthetic and metabolic pathways with AEA and 2-AG, enhancing endocannabinoid activity via the “entourage effect” (Mock et al., 2023; Castonguay-Paradis et al., 2020; Anand et al., 2022). Additionally, these mediators interact with various channels and receptors, including transient receptor potential (TRP) channels, orphan G protein-coupled receptors (orphan GPRs), and peroxisome proliferator-activated receptors α and γ (PPARα and γ) (Di Marzo, 2018). The eCBome plays a crucial role in cellular signaling and physiological regulation (**Figure 1**).

Recent studies highlight strong gut microbiota–eCBome associations. Germ-free (GF) mice exhibit significant changes in the expression of eCBome components such as CB1, GPR18, GPR55, and PPARα. These changes vary across different gut regions compared to conventionally housed mice. Additionally, AEA and 2-AG levels in the brain show age- and sex-related variations. These changes were partially reversed in GF mice through fecal microbiota transplantation, reinstating normal eCBome function (Manca et al., 2020a; Manca et al., 2020b). Human studies also report correlations between eCBome plasma components and gut bacterial genera, particularly those involved in maintaining gut barrier integrity. Specifically, AEA and related N-acylethanolamine plasma levels are positively correlated with Faecalibacterium and Akkermansia, but negatively correlated with Bifidobacterium. Conversely, 2-AG and other monoacylglycerols are positively correlated with Parabacteroides and Odoribacter, and negatively correlated with Prevotella (Ortiz-Alvarez et al., 2022). AEA has been shown to reverse dysbiosis in the gut and lungs in conditions like acute respiratory distress syndrome, promoting beneficial short-chain fatty acid (SCFA)-producing bacteria and reducing inflammation in both the gut and lungs (Sultan et al., 2021).

Despite recent advances, our understanding of gut microbiota–eCBome interactions remains limited. Research is constrained by differences between animal models and human physiology, inconsistencies in health and disease models, and insufficient mechanistic exploration. Additionally, many studies focus solely on particular health or disease states, overlooking the dynamic interactions between the microbiota and the eCBome across different physiological and pathological contexts. The exploration of these mechanisms is often superficial, with detailed molecular mechanisms inadequately elucidated. These limitations hinder our comprehensive understanding of how interactions between the gut microbiota and the eCBome impact host health.

**Figure 2:**
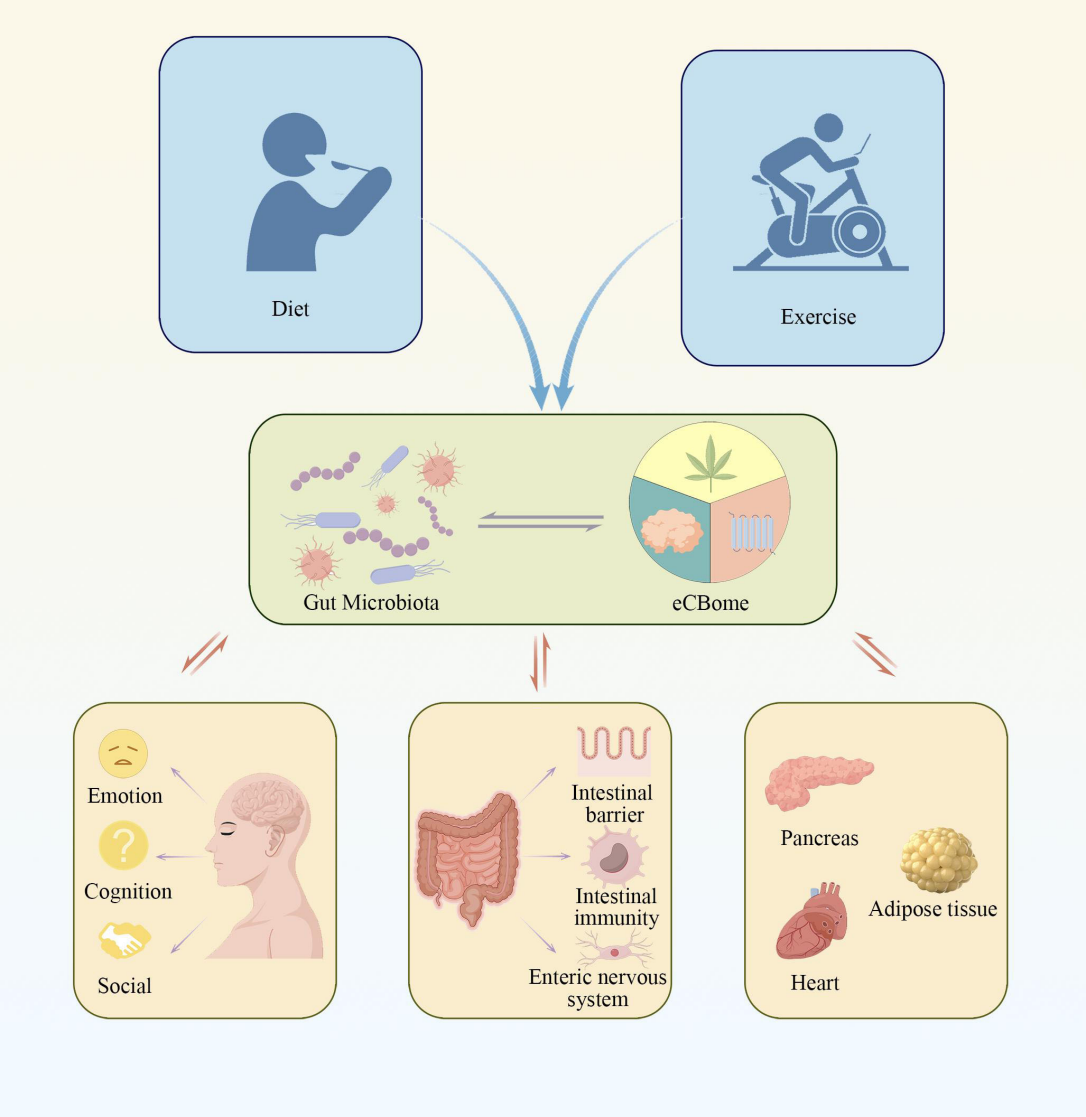
The figure illustrates the complex signaling pathways between the gut microbiota and the endocannabinoidome, highlighting how these interactions are regulated by various lifestyle factors. This interplay significantly impacts the host’s metabolic, immune, and neural functions. Such mechanisms may play a crucial role in the development of various diseases.

Therefore, in this review, we systematically summarize the complex interactions between the gut microbiota and the eCBome, to provide a thorough evaluation of existing research and a critical analysis of knowledge gaps in the literature (**Figure 2**). By synthesizing current findings, this review not only lays theoretical foundations for a deeper understanding of the interactions between the eCBome and the gut microbiota, but also provides valuable scientific insights for future research directions.

## Dietary influences on the eCBome and the gut microbiota

Dietary habits have a rapid and direct impact on the human gut microbiota and the eCBome (Bourdeau-Julien et al., 2023). Research has shown that overall dietary patterns influence gut microbiota composition and eCBome mediator levels more strongly than individual macronutrient intake. Foods like vegetables, refined grains, and olive oil are key factors that affect circulating NAE levels. This specific dietary pattern is also associated with the relative abundance of Clostridiaceae, Peptostreptococcaceae, and Veillonellaceae families in the gut microbiota (Castonguay-Paradis et al., 2023). These findings suggest that dietary patterns may regulate eCBome mediator activity by altering gut microbiota composition, thereby influencing host health. However, current research often focuses on short-term effects, with long-term effects and underlying mechanisms remaining inadequately explored. Furthermore, the generalizability of these findings across different populations still needs validation.

A high-fat diet significantly alters the gut microbiota and modulates eCBome function through complex interactions with specific bacterial populations. The impact of different dietary fat types on the gut microbiota and eCBome varies notably. For example, a diet high in olive oil reduces Lachnospiraceae_UCG006 abundance while increasing Parasutterella and Faecalibaculum levels. These microbial changes may influence host metabolism through metabolic pathway reprogramming. N-oleoylalanine, an endocannabinoid analog, increases Akkermansia and Marvinbryantia abundance in high-lard diets, which is associated with metabolic improvements and shows potential for weight management (Forte et al., 2023). High-fat diets also affect eCBome and gut microbiota in ways modulated by other dietary components, such as proteins. High-fat diets increase CB1 receptor expression and triglyceride accumulation while decreasing gut microbiota diversity. This is accompanied by increased Firmicutes abundance and decreased levels of Deferribacteres and Verrucomicrobia. Additionally, protein intake from beef and chicken exacerbates these effects (Ijaz et al., 2020).

The impact of a high-fat high-sugar (HFHS) diet on the gut microbiota and the eCBome is complex and dynamic. In the short term, an HFHS diet leads to a rapid decrease in gut microbiota diversity, especially in the cecum, with notable reductions in Barnesiella and Eubacterium abundance, and increased Clostridium sensu stricto levels. This diversity loss appears to recover over time, likely due to microbiota restructuring. An HFHS diet also significantly alters eCBome mediator levels in the ileum and plasma. For instance, AEA levels in the ileum rise significantly within 10 days, while PEA and OEA levels decrease early on, with PEA returning to normal by day 56. In plasma, AEA and 2-AG levels increase significantly at days 10 and 21, but decrease sharply by day 56, while OEA, 2-OG, and omega-3-derived eCBome mediators generally decrease. These changes are closely associated with decreased FAAH enzyme expression and variations in Adlercreutzia and Barnesiella abundance (Lacroix et al., 2019). Thus, an HFHS diet regulates eCBome function through changes in relevant metabolic enzyme expression and microbiota composition. Additionally, a selenium-rich diet provides anti-inflammatory effects under the HFHS diet, mitigating metabolic disturbances by modulating eCBome mediator levels and improving gut microbiota composition (Agudelo et al., 2022). Overall, the effects of an HFHS diet on the eCBome and gut microbiota are complex, underscoring the importance of exploring their regulatory mechanisms and long-term health impacts.

The Mediterranean diet, characterized by abundant vegetables, fruits, legumes, nuts, whole grains, fish, and monounsaturated fatty acids, significantly alters eCBome mediator levels in the short term. Research indicates that this dietary pattern notably increases NAE and 2-MAG levels derived from ω-3 fatty acids and oleic acid, which positively affect metabolic health (Castonguay-Paradis et al., 2020). The diet is also associated with increased relative Christensenellaceae abundance in the gut and may be negatively correlated with obesity risk (Garcia-Mantrana et al., 2018), suggesting regulatory effects on the gut microbiota. However, a study by Castonguay-Paradis et al. found that Christensenellaceae abundance was positively correlated with whole grain intake but negatively correlated with LEA and PEA plasma levels. This effect was not significant after adjusting for fat quality (Castonguay-Paradis et al., 2023). Thus, the relationship between the Mediterranean diet and the gut microbiota may be more complex than currently understood, potentially influenced by multiple factors, including dietary components and individual metabolic states. Transitioning from a standard to a Mediterranean diet decreases 2-AG plasma levels and increases OEA and PEA levels, along with an increase in the beneficial bacterium Akkermansia muciniphila(A. muciniphila) (Tagliamonte et al., 2021). These changes suggest that the Mediterranean diet may influence overall health by modulating eCBome mediator levels and improving gut microbiota composition.

Furthermore, some studies suggest that interactions between certain microbiota and eCBome mediators may occur independently of dietary factors. For instance, the Peptostreptococcaceae and Veillonellaceae families showed positive correlations with multiple NAEs, while the Akkermansiaceae family was negatively correlated with 2-MAGs. These relationships remained significant even after adjusting for fat quality (Castonguay-Paradis et al., 2020), indicating that gut microbiota influences the eCBome system through mechanisms independent of diet. Consequently, the complex interactions between the gut microbiota and the eCBome system require further investigation to elucidate their specific mechanisms and provide a theoretical basis for personalized dietary interventions. This could lead to more precise health management strategies.

## Interactions between exercise, eCBome, and the gut microbiota

Regular physical activity is widely recommended in contemporary health research as an effective strategy for reducing disease burden. For example, the World Health Organization suggests engaging in 150 minutes of moderate-intensity physical activity per week to maintain overall health (Bull et al., 2020). Rojas-Valverde et al. found that regular exercise promoted gut microbiota diversity and stability. It also increased the abundance of specific bacterial species, especially those linked to butyrate production. These changes were not significantly influenced by diet, age, weight, fat percentage, energy intake, or fiber intake (Estaki et al., 2016; Munukka et al., 2018; Rojas-Valverde et al., 2023).

The eCBome undergoes significant changes during physical activity. Prolonged high-intensity exercise can induce “runner’s high,” a state associated with the release of eCBome mediators. This release contributes to feelings of euphoria and satisfaction. The gut microbiota influences this phenomenon, as metabolites produced by gut microbes stimulate sensory neurons expressing CB1 receptors. These neurons transmit exercise signals to the brain, enhancing dopamine levels and improving exercise performance (Dohnalová et al., 2022). Additionally, exercise may increase endogenous cannabinoid levels. This could shift the gut microbiota toward bacteria that produce short-chain fatty acids (SCFAs), thus boosting SCFA production without dietary changes (Vijay et al., 2021). These interactions between the gut microbiota and the eCBome help explain how exercise can benefit overall health.

## The role of eCBome-gut microbiota interactions in gastrointestinal disorders

A. muciniphila is a key bacterium in regulating intestinal barrier function. Studies with the CB1 receptor antagonist (SR141716A) have shown that this antagonist improves colonic barrier function and increases A. muciniphila abundance (Mehrpouya-Bahrami et al., 2017). This suggests a potential interaction between A. muciniphila and the CB1 receptor. The receptor may influence A. muciniphila abundance by modulating the intestinal microenvironment. A. muciniphila’s impact on intestinal barrier function involves complex regulation of CB1 and CB2 receptors, as well as PPAR pathways. In studies using a human colorectal adenocarcinoma cell line, active A. muciniphila and its outer membrane vesicles significantly inhibited CB1 and CB2 receptor mRNA expression at high concentrations. Conversely, inactivated A. muciniphila increased receptor expression at medium to low concentrations. Notably, FAAH expression was significantly upregulated under all experimental conditions, and PPAR expression was also increased under various interventions (Ghaderi et al., 2022).The contrasting effects of active and inactivated A. muciniphila suggest that its regulatory mechanisms may vary depending on bacterial state and dosage, possibly involving specific cellular signaling pathways or metabolic products. Further research is needed to clarify these mechanisms and better define A. muciniphila’s roles in different inflammatory environments.

PEA, a member of the eCBome, exhibits significant anti-inflammatory, antioxidant, and immunomodulatory effects in various gastrointestinal diseases (Branković et al., 2024). Research has shown that PEA reduces high-fat diet-induced dysbiosis by increasing beneficial bacteria like Bifidobacterium, Oscillospiraceae, and the butyrate-producing bacterium Turicibacter sanguinis. This helps reduce inflammation and improves mucosal barrier integrity (Pirozzi et al., 2023). PEA’s anti-inflammatory effects are mediated through PPARα activation, which reduces inflammatory mediator release. By inhibiting T-helper 1 and T-helper 17 cell inflammatory responses, increasing interleukin-22 production, and promoting antimicrobial peptide synthesis (e.g., α-defensins), PEA regulates small intestinal microbiota composition, including Bacteroidetes, Proteobacteria, Firmicutes, and Actinobacteria. These actions contribute to maintaining gut microbiota health and influencing the intestinal microenvironment (Salzman et al., 2010; Manoharan et al., 2016). However, the specific mechanisms underlying PEA and PPARα’s interactions with the gut microbiota and their effects under various pathological conditions need further investigation.

In a study using an inflammatory bowel disease (IBD) model, certain NAEs like LEA and AEA promoted the growth of IBD-associated species such as Escherichia coli and Ruminococcus gnavus, while inhibiting IBD-depleted species like Bacteroides cellulosilyticus and Fibrobacter succinogenes. Conversely, PEA had minimal effects on these species. In ex vivo experiments with intestinal microbiota, specific NAE combinations were shown to shift the microbial community from a healthy to an IBD-like state (Fornelos et al., 2020). These findings are based on associative studies, which have not yet established direct causal relationships between elevated NAE levels and microbial community changes.

Interactions between endogenous cannabinoids and microbial metabolites are becoming clearer. Butyrate, a primary energy source for colonocytes, alleviates inflammation by regulating inflammatory pathways, inhibiting T-helper 17 cells, and increasing regulatory T cell proportions. This highlights the important role of SCFAs in intestinal health. Exercise studies have found positive correlations between AEA and SCFA-producing bacteria like Bifidobacteria, Coprococcus 3, and Enterococcus faecalis, with a particularly strong correlation with butyrate. This suggests that SCFA’s anti-inflammatory effects may be partially mediated through the ECS (Vijay et al., 2021). However, specific mechanisms require thorough investigation. Additionally, Faecalibacterium prausnitzii, a butyrate-producing bacterium, significantly increased expression of tight junction proteins (zonula occludens-1 and occludin) and upregulated various PPAR subtypes via its exosomes (Moosavi et al., 2020). Future research should explore how the ECS regulates F. prausnitzii metabolites and assess their impact on host inflammatory responses, particularly the effects of ECS deficiency or overexpression on F. prausnitzii functionality and SCFA production, to better understand interactions between these factors.

Visceral hypersensitivity is a common feature in functional gastrointestinal disorders and IBD. Early studies suggested that oral Lactobacillus acidophilus administration to rodents might alleviate visceral pain by upregulating CB2 receptor expression (Rousseaux et al., 2007). Later research by Aguilera et al. supported this, showing that antibiotic treatment increased Lactobacillus abundance, upregulated CB2 receptor expression, and reduced visceral pain (Aguilera et al., 2015). However, these findings may be confounded by the complex effects of antibiotics on the gut microbiota, and clinical trials have produced inconsistent results (Ringel-Kulka et al., 2014). This variability highlights the need for future research to explore specific regulatory mechanisms of L. acidophilus on CB2 receptors and to validate these actions in clinical trials.

The interactions between the eCBome and the gut microbiota are crucial for maintaining intestinal health, particularly in regulating intestinal barrier function and managing related diseases. Although current research provides valuable insights, many mechanisms remain unclear, especially regarding the interactions between NAEs and the microbiota, as well as the regulation of SCFAs. Future research should focus on establishing causal relationships between the eCBome and the microbiota, and assessing the clinical implications of these findings, particularly in the context of various pathological conditions.

## The eCBome and the gut microbiota in neurobehavioral and emotional regulation

The eCBome plays essential roles in regulating neurobehavioral function, emotions, and stress responses through its interactions with the hypothalamic-pituitary-adrenal (HPA) axis, the vagus nerve, and neurotransmitter-hormone regulation along the gut-brain axis (Gunther et al., 2024). Research has shown that antibiotic-induced dysbiosis leads to depressive-like behaviors and social deficits in mice, which are closely associated with reduced gut microbiota diversity and increased abundance of phyla such as Proteobacteria and Actinobacteria (Guida et al., 2018). Probiotic supplementation not only restored the gut microbiota balance but also alleviated depressive symptoms by modulating the levels of N-arachidonoylserotonin, N-oleoylserotonin, and AEA in the jejunum. Additionally, chronic mild stress models have shown that depressive-like behavior and impaired neurogenesis can be transmitted between mice via fecal microbiota transplantation, accompanied by 2-AG precursor deficiency in serum, reduced hippocampal 2-AG levels, and disruption of CB1-mammalian target of rapamycin (mTOR) signaling. Lactobacillus plantarum supplementation also restored these neurotransmitter levels and improved depressive-like behaviors (Chevalier et al., 2020). While these animal studies have provided valuable insights into the interactions between the eCBome and gut microbiota in mental disorders, differences in probiotic species and their mechanisms require further clinical validation.

The TwinsUK human cohort study identified a negative correlation between gut microbiota diversity and PEA levels, as well as depressive symptoms. Specific microbial genera, such as Blautia and Dorea, showed associations with PEA levels and depressive symptoms (Minichino et al., 2021). However, individual variability and the detailed mechanisms need further investigation.

In autism spectrum disorders (ASD), interactions between the eCBome and the gut microbiota show significant therapeutic potential. PEA has been shown to reverse autism-like behaviors in animal models, potentially by ameliorating gut microbiota dysbiosis (Cristiano et al., 2018). Butyrate and propionate are also implicated in ASD regulation. Butyrate may reduce key endocannabinoid (eCB) synthesis, via AEA and 2-AG, by influencing cannabinoid synthesis and degradation enzyme levels in intestinal epithelial cells. This aligns with the recorded eCB reductions in children with ASD (Karhson et al., 2018; Aran et al., 2019; Hwang et al., 2021). Moreover, increased propionate-producing bacteria may be linked to ASD-like behaviors. CB1 receptor antagonism altered gut microbiota composition and enhanced propionate production, which was associated with changes in ASD symptoms (Mehrpouya-Bahrami et al., 2017). Although these studies provide valuable insights, further clinical validation is required to assess their applicability and effectiveness.

Neuropathological changes and cognitive impairments associated with Alzheimer’s Disease (AD) and alcohol use disorder (AUD) may be influenced by interactions between the gut microbiota and the ECS. eCBs, particularly AEA, can increase intestinal permeability, leading to a “leaky gut” and allowing endotoxins, such as lipopolysaccharide (LPS), to enter the bloodstream. This can trigger chronic central nervous system inflammation through the vagus nerve and gut endocrine signals, potentially exacerbating AD progression (Cani et al., 2016). In AD patients’ brain tissue, excessive CB2 receptor and FAAH expression was associated with amyloid β deposition. CB1 receptor activation may exert neuroprotective effects by reducing amyloid β deposition and tau phosphorylation (Benito et al., 2003; Esposito et al., 2006). In a mouse model of cognitive impairment induced by chronic neuropathic pain, both gut microbiota and ECS dysregulation were closely related to cognitive deficits, with diurnal variations (Hua et al., 2021; He et al., 2024). The detrimental effects of alcohol on intestinal permeability and microbiota may impact the brain and behavior through neurological and inflammatory pathways. This can promote relapse, addictive behaviors, and cognitive, emotional, and behavioral issues related to AUD. The ECS may exert protective effects by regulating intestinal permeability and the microbiota, and plays a critical role in alcohol-induced gut inflammation (Karoly et al., 2020).

## Interactions between the gut microbiota and the eCBome in metabolic disorders

The eCBome plays key roles in regulating energy metabolism and impacts multiple organs, including the brain, liver, adipose tissue, muscle, and pancreas. This regulation affects gastrointestinal function, inflammatory responses, appetite, satiety, and postprandial blood glucose levels. Several studies have emphasized the importance of eCBome and gut microbiota interactions, particularly in the context of obesity and related metabolic disorders (Di Marzo et al., 2008; Izzo et al., 2009; Piomelli, 2013; DiPatrizio and Piomelli, 2015).

In obesity and diabetes genetic models, specific changes in eCBome molecules have been linked to gut bacterial interactions. In genetically obese mice, elevated OEA levels in the liver were associated with dysregulated fat metabolism. In contrast, genetically diabetic mice showed reduced 2-OG levels in subcutaneous fat and decreased AEA levels in visceral fat, indicating metabolic disturbances. Gut microbiota analyses revealed correlations between Clostridium sensu stricto 1 in the gut and metabolic molecules in adipose tissue, suggesting the microbiota’s influence on the eCBome (Suriano et al., 2022). Research in obese women also found that capsaicin, an eCBome analog, improved body composition and metabolic health by increasing Clostridia and Flavonifractor abundance in the gut (Manca et al., 2021).

In diet-induced obesity mouse models, interactions between the gut microbiota and ECS were observed. ECS activation promoted adipogenesis, while the Gram-negative bacterial metabolite LPS affected fat metabolism by inducing adipogenesis through CB1 receptor blockade, which may have indirectly contributed to atherosclerosis (Muccioli et al., 2010; Moludi et al., 2018). Probiotic and prebiotic interventions improved gut microbiota balance and positively impacted cardiovascular risk factors, possibly through modulating CB2 receptor expression (Liu et al., 2023). In diabetic mouse models induced by a high-fat, high-sugar diet combined with streptozotocin, gut microbiota imbalance and abnormal ECS activation, particularly through CB1 receptors, were closely associated with metabolic disorders and cognitive impairments (Hussein et al., 2022).

Overall, the eCBome plays a vital role in regulating energy balance across different organs, influencing gastrointestinal function and metabolic processes. The gut microbiota also significantly impacts energy metabolism through its metabolites and regulatory effects on the eCBome. While these interactions have been extensively studied, many underlying mechanisms remain unclear. Further research is needed to address how the microbiota regulates eCBome functions, whether these effects apply to different individuals, and the long-term outcomes of such interventions. These questions should be explored in basic research and larger-scale, long-term clinical studies.

## Molecular mechanisms and future directions for eCBome-gut microbiota interactions

Recent functional metagenomics research identified commenda-mide, a long-chain N-acylamide with GPR activity encoded by the human microbiota, suggesting structural convergence between human signaling molecules (e.g., eCBs) and microbiota-encoded metabolites (Cohen et al., 2015). Specifically, N-acylamide synthase genes are enriched in symbiotic bacteria, and the lipids they encode interact with GPRs in a manner similar to eCBs. Chang et al. reported that Eubacterium rectale produces OEA by catalyzing the conjugation of oleic acid and ethanolamine via its endogenous biosynthetic gene cluster (Chang et al., 2021). Conversely, the impact of the eCBome on the gut microbiota also warrants further exploration. For instance, 2-AG not only directly inhibits pathogen virulence programs and reduces infection severity, but also antagonizes bacterial quorum-sensing receptor E, preventing activation of the pathogen type III secretion system and interfering with pathogen survival and proliferation, thus providing effective resistance against gut infections caused by Enterobacteriaceae pathogens (Ellermann et al., 2020).

## Limitations

While growing evidence suggests that interactions between the gut microbiota and the eCBome are influenced by various lifestyle factors and disease models, the precise molecular mechanisms behind these interactions remain unclear. Most studies to date have focused on observational phenotypes and correlation analyses, which do not provide direct evidence of causality. As a result, these studies limit our comprehensive understanding of the molecular mechanisms involved. Current disease models are often limited by small sample sizes and focus on specific diseases or populations. Consequently, findings often lack generalizability across diverse disease types and broader populations. Variations in research methodologies—such as experimental design, sample sources, technological platforms, and data analysis techniques—have led to heterogeneous results, complicating the comparison of findings across studies. Future research should expand sample sizes to include more diverse disease types and populations while standardizing methodologies to improve result comparability.

## Future directions

Future research should move from broad correlational studies to more detailed mechanistic investigations aimed at clarifying the molecular interactions between the microbiota and the eCBome. This includes exploring how microbiota and its metabolites influence eCBome functions and identifying the associated signaling pathways and enzyme systems. High-throughput sequencing technologies, such as metagenomic sequencing, should be employed to identify the microbiota. These technologies can also be used to identify its metabolites. These findings can be supported by mass spectrometry and nuclear magnetic resonance technologies to analyze the regulatory effects of these metabolites on the eCBome. Furthermore, transcriptomics and proteomics can help identify signaling pathways and enzymes, with subsequent validation in cell-based assays or animal models. Additionally, investigating changes in microbiota–eCBome interactions under different disease conditions, using disease models and clinical samples, could provide more clinically relevant data. Integrating data from various technological approaches, combined with microbiology, pharmacology, and bioinformatics, will advance this field, enhance reproducibility and reliability, and provide new theoretical and practical foundations for disease prevention, diagnosis, and treatment.
